# Effects of Contemporary Therapies on Cardiovascular and Renal Outcomes in Diabetic Kidney Disease: A Systematic Review of Randomized Controlled Trials (RCTs)

**DOI:** 10.7759/cureus.95400

**Published:** 2025-10-25

**Authors:** Wisam Bachar Al Sumodi, Muslim Sajjad, Sami Ullah Rana, Ghulam Ahmed, Aakash Hans, Waleed Mirani

**Affiliations:** 1 Internal Medicine, Ivane Javakhishvili Tbilisi State University, Tbilisi, GEO; 2 Internal Medicine, Indus Medical College and Hospital, Tando Muhammad Khan, PAK; 3 Internal Medicine, Liaquat University of Medical and Health Sciences, Jamshoro, PAK; 4 Research, Henry Ford Health System, Detroit, USA; 5 Internal Medicine, White River Health System, Batesville, USA; 6 Internal Medicine, Nishtar Medical University, Multan, PAK

**Keywords:** cardiovascular outcomes, diabetic kidney disease, finerenone, hyperkalemia, mineralocorticoid receptor antagonist, renal outcomes, type 2 diabetes

## Abstract

This systematic review evaluated the impact of contemporary therapies on cardiovascular (CV) and renal outcomes in patients with diabetic kidney disease (DKD), with a particular focus on finerenone as a novel nonsteroidal mineralocorticoid receptor antagonist. Across eight eligible studies involving more than 13,000 participants with type 2 diabetes and chronic kidney disease (CKD), finerenone consistently reduced the risk of composite CV events, including CV death, myocardial infarction, stroke, and hospitalization for heart failure, as well as composite renal outcomes such as kidney failure, sustained estimated glomerular filtration rate decline, or renal death. Subgroup analyses demonstrated that these benefits extended across diverse populations, including patients with stage 4 CKD, those stratified by age and sex, and individuals receiving diuretics, GLP-1 receptor agonists, or with baseline left ventricular hypertrophy. Importantly, the renal protective effects appeared dose-dependent up to 20 mg daily, with safety concerns primarily limited to an increased incidence of hyperkalemia, though discontinuation rates remained low. An earlier comparative trial of renin-angiotensin system inhibitors showed no significant differences between agents but highlighted tolerability advantages with angiotensin receptor blockers. Collectively, these findings reinforce the role of finerenone as an effective therapy for cardiorenal risk reduction in DKD, while also identifying areas for further research including advanced kidney disease, long-term renal preservation, and combination therapy strategies.

## Introduction and background

Diabetic kidney disease (DKD) is one of the most common and devastating complications of type 2 diabetes mellitus (T2DM), representing the leading cause of chronic kidney disease (CKD) and end-stage renal disease (ESRD) worldwide [1]. Globally, DKD affects nearly 40% of patients with diabetes, contributing to increased morbidity, mortality, and healthcare costs [2]. Beyond progressive renal dysfunction, DKD substantially elevates the risk of adverse cardiovascular (CV) events, including heart failure, myocardial infarction (MI), and stroke. This dual burden underscores DKD as both a renal and CV disorder, where outcomes are tightly interlinked [3]. The underlying pathophysiological connection arises from shared mechanisms such as endothelial dysfunction, chronic inflammation, oxidative stress, and neurohormonal activation, which accelerate both renal and myocardial injury, ultimately amplifying overall CV risk.

For decades, inhibition of the renin-angiotensin-aldosterone system (RAAS) with angiotensin-converting enzyme inhibitors or angiotensin receptor blockers (ARBs) formed the cornerstone of therapy to slow CKD progression and reduce CV risk in DKD [4]. However, despite RAAS blockade, many patients continued to experience residual risk of kidney failure and CV complications. The emergence of sodium-glucose cotransporter-2 (SGLT2) inhibitors and glucagon-like peptide-1 receptor agonists (GLP-1 RAs) has reshaped the therapeutic paradigm, demonstrating benefits beyond glycemic control, particularly in kidney protection and CV risk reduction [5]. Importantly, these agents may exert complementary or synergistic effects, as SGLT2 inhibitors predominantly reduce intraglomerular pressure and volume overload, while GLP-1 RAs modulate endothelial inflammation, weight, and atherogenesis-together contributing to holistic cardiorenal protection.

More recently, finerenone, a novel non-steroidal mineralocorticoid receptor antagonist (MRA), has been shown to further reduce both kidney and CV events when added to standard of care [6]. Pivotal phase III trials have confirmed the efficacy and safety of finerenone across a spectrum of DKD severity. Additionally, subgroup analyses have provided insights into its effectiveness in patients with advanced CKD, comorbid atherosclerotic CV disease, and in those receiving concomitant therapies such as diuretics and GLP-1 RAs [7].

Despite significant advances, there remains uncertainty regarding the magnitude and consistency of benefit from these contemporary therapies across diverse patient populations with DKD [8]. Moreover, social determinants of health, such as disparities in access to healthcare, socioeconomic status, and education, further influence CV and renal outcomes, often magnifying disease burden in underserved populations. Questions also remain about how therapies such as finerenone, SGLT2 inhibitors, GLP-1 RAs, and RAAS inhibitors compare or complement one another in improving both kidney and CV outcomes. Direct quantitative head-to-head comparisons across these classes were not attempted in this review; instead, a narrative synthesis was conducted to integrate evidence from high-quality randomized trials and subgroup analyses. Synthesizing data from these studies is essential to clarify their role in clinical practice and inform guideline recommendations.

The objective of this systematic review is to critically evaluate and synthesize evidence from contemporary clinical trials and subgroup analyses assessing the effects of novel and established therapies on CV and renal outcomes in patients with DKD. By integrating data from finerenone, RAAS inhibition, and adjunctive therapeutic strategies, this review aims to provide a comprehensive overview of the evolving treatment landscape and highlight future directions for optimizing patient care.

## Review

Materials and methods

Study Design and Protocol

This systematic review was conducted in accordance with the Preferred Reporting Items for Systematic Reviews and Meta-Analyses (PRISMA) guidelines [9]. The review protocol was developed prior to data collection to ensure transparency and methodological rigor. Our primary objective was to evaluate the effects of contemporary therapies, with a focus on finerenone and renin-angiotensin system inhibitors, on CV and renal outcomes in patients with DKD.

Eligibility Criteria (PICO Framework)

The research question was framed using the PICO methodology [10]. The study population (P) included adult patients with T2DM and chronic kidney disease, defined by reduced estimated glomerular filtration rate (eGFR) and/or albuminuria. The interventions (I) of interest were contemporary pharmacological therapies targeting cardiorenal risk, primarily finerenone, as well as renin-angiotensin system inhibitors and adjunctive agents such as SGLT2 inhibitors or GLP-1 receptor agonists when assessed in conjunction with finerenone. Comparators (C) included placebo, standard of care alone, or alternative pharmacological regimens. The primary outcomes (O) were composite CV events, including CV death, nonfatal MI, nonfatal stroke, and hospitalization for heart failure, as well as composite renal outcomes, including kidney failure, sustained eGFR decline of ≥57%, or renal death. Because renal composite definitions varied across trials (e.g., sustained eGFR decline of ≥40% vs ≥57% and/or kidney failure), we extracted each trial’s prespecified renal composite and reported it verbatim; for cross-trial interpretability in the narrative synthesis, we labeled these uniformly as “trial-defined kidney composite”. Where both thresholds were reported within a trial, we preferentially cited the more conservative (≥57%) threshold in-text and cross-checked conclusions against the alternative threshold in sensitivity statements. Secondary outcomes included changes in albuminuria, rates of hyperkalemia, and treatment discontinuations.

Search Strategy and Study Selection

A comprehensive search of PubMed, Embase, and Cochrane Central Register of Controlled Trials (CENTRAL) was performed to identify eligible studies published up to May 2025. Searches were limited to human studies, adults (≥18 years), randomized controlled trials (RCTs), and English-language publications. The search terms combined medical subject headings (MeSH/Emtree) and keywords related to “finerenone,” “mineralocorticoid receptor antagonists,” “diabetic kidney disease,” “type 2 diabetes,” “cardiovascular outcomes,” and “renal outcomes.” Boolean operators were applied to maximize sensitivity, and filters were applied to restrict results to RCTs and high-quality clinical analyses. In paragraph form, an example PubMed strategy was: (“Diabetic Kidney Disease” OR “diabetic nephropathy” OR “diabetes” AND “chronic kidney disease”) AND (finerenone OR “nonsteroidal mineralocorticoid receptor antagonist” OR “mineralocorticoid receptor antagonists” OR spironolactone OR eplerenone OR “SGLT2 inhibitor” OR empagliflozin OR dapagliflozin OR canagliflozin OR “GLP-1 receptor agonist” OR liraglutide OR semaglutide) AND (randomized OR randomly OR placebo) AND (cardiovascular OR renal OR kidney OR albuminuria). Similar logic was adapted for Embase/CENTRAL with appropriate subject headings.

Gray literature sources (reference lists of eligible studies/reviews) were screened, and ClinicalTrials.gov was queried for ongoing or unpublished RCTs; preprints were not included in the final analysis. After duplicate removal, titles and abstracts were independently screened by two reviewers, followed by full-text evaluation for eligibility. Disagreements at either stage were resolved by consensus or a third reviewer.

Data Extraction and Synthesis

Data were extracted systematically into predesigned forms, capturing study characteristics, patient demographics, interventions, comparators, follow-up duration, and CV and renal outcomes. Where available, hazard ratios (HRs) and 95% confidence intervals (CIs) were collected. Two reviewers independently and in duplicate extracted all data and assessed risk of bias; a third reviewer adjudicated discrepancies to minimize extraction bias. A narrative synthesis was performed given the heterogeneity of trial designs and subgroup analyses. Quantitative head-to-head comparative analyses across drug classes were not attempted; the review integrates evidence descriptively from randomized trials and subgroup/post hoc analyses. Special emphasis was placed on consistency across prespecified and post hoc analyses, the applicability of findings to diverse subgroups (e.g., age, sex, diuretic or GLP-1RA use, stage 4 CKD), and safety signals such as hyperkalemia. When renal endpoints used different decline thresholds (≥40% vs ≥57%), results were summarized using each study’s trial-defined composite, with threshold details explicitly reported in tables and referenced in-text for clarity.

Quality Assessment

The risk of bias was independently assessed using the Cochrane Risk of Bias tool [11] for RCTs, evaluating domains including sequence generation, allocation concealment, blinding, incomplete outcome data, and selective reporting. For subgroup and post hoc analyses, considerations of indirectness and imprecision were applied. The overall certainty of evidence for each primary outcome was graded using the GRADE (Grading of Recommendations, Assessment, Development, and Evaluation) framework [12].

Statistical Considerations

Because of the diversity in design and endpoints across included studies, a quantitative meta-analysis was not performed. Instead, findings were synthesized descriptively, with HRs and event rates presented in structured tables. Emphasis was placed on the consistency of direction and magnitude of treatment effects, as well as subgroup interactions. Where possible, absolute risk reductions and numbers needed to treat were calculated from reported trial data to facilitate clinical interpretation.

Results

Study Selection Process

**Figure 1:**
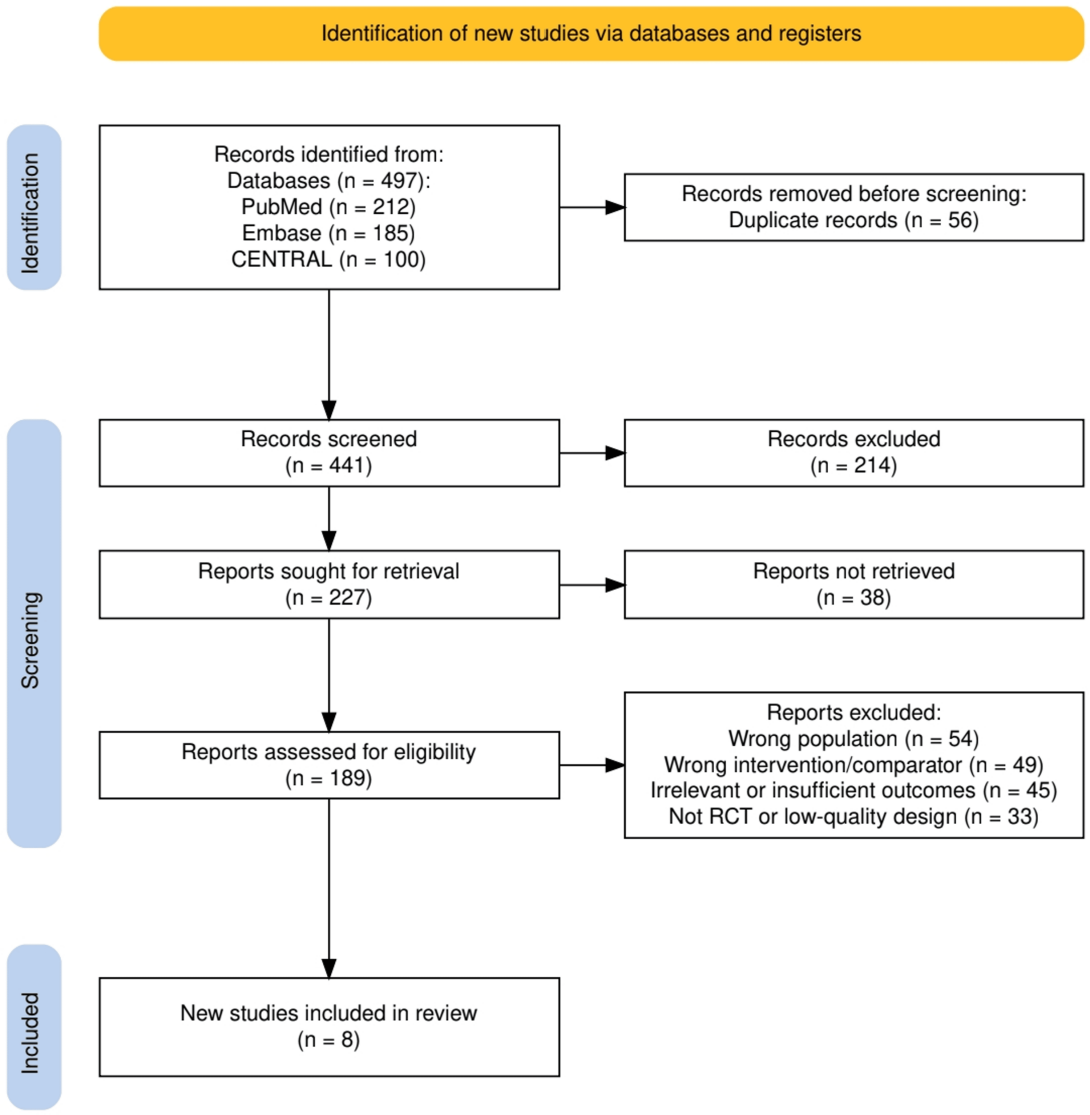
The PRISMA flow chart represents the study selection process. PRISMA: Preferred Reporting Items for Systematic reviews and Meta-Analyses

Figure 1 provides a visual overview of the study selection process according to PRISMA guidelines. A total of 497 records were initially retrieved from PubMed (n = 212), Embase (n = 185), and CENTRAL (n = 100). After the removal of 56 duplicate records, 441 unique titles and abstracts were screened. Of these, 214 were excluded due to irrelevance to the research question. The remaining 227 reports were sought for retrieval, with 38 not accessible. A total of 189 full-text articles were assessed for eligibility, of which 181 were excluded for reasons such as wrong population (n = 54), inappropriate intervention or comparator (n = 49), irrelevant or insufficient outcomes (n = 45), and non-randomized or low-quality study design (n = 33). Ultimately, eight RCTs met the inclusion criteria and were included in the final synthesis, ensuring that the evidence base reflected high-quality, clinically relevant data.

Characteristics of the Selected Studies

Table 1 summarizes the key characteristics of the eight studies included in this review, highlighting their design, patient populations, interventions, and primary outcomes. The majority were large, multicenter, RCTs or prespecified pooled analyses involving over 13,000 patients with type 2 diabetes (T2D) and CKD. Most studies evaluated finerenone in addition to optimized renin-angiotensin system inhibition, compared with placebo plus standard of care, with median follow-up durations ranging from 2.6 to 3 years. The primary CV outcomes consistently included a composite of CV death, MI, stroke, and hospitalization for heart failure, while renal outcomes were generally defined as kidney failure, sustained decline in estimated glomerular filtration rate of at least 57%, or renal death. Across the trials, finerenone demonstrated consistent reductions in both CV and renal events, with added benefits in subgroups such as those with left ventricular hypertrophy (LVH) or concomitant GLP-1 receptor agonist use. However, hyperkalemia emerged as a recurrent safety concern, though discontinuation rates remained low. One earlier trial comparing ACE inhibitors, ARBs, and combination therapy found no significant differences in cardiorenal outcomes, though ARB monotherapy was better tolerated. Collectively, the evidence presented in Table 1 underscores the robust clinical potential of finerenone, while also identifying areas of heterogeneity that warrant further investigation.

**Table 1:** Characteristics of the selected randomized controlled trials and subgroup analyses of contemporary therapies in diabetic kidney disease. RCT: Randomized controlled trial; FIDELIO-DKD: Finerenone in Reducing Kidney Failure and Disease Progression in Diabetic Kidney Disease; FIGARO-DKD: Finerenone in Reducing Cardiovascular Mortality and Morbidity in Diabetic Kidney Disease; T2D: Type 2 diabetes; CKD: Chronic kidney disease; eGFR: Estimated glomerular filtration rate; RAAS: Renin–angiotensin–aldosterone system; CV: Cardiovascular; MI: Myocardial infarction; HF: Heart failure; HR: Hazard ratio; CI: Confidence interval; IQR: Interquartile range; LVH: Left ventricular hypertrophy; ECG: Electrocardiogram; GLP-1RA: Glucagon-like peptide-1 receptor agonist; UACR: Urinary albumin-to-creatinine ratio; PK: Pharmacokinetics; SGLT2: Sodium–glucose cotransporter-2; ACEi: Angiotensin-converting enzyme inhibitor; ARB: Angiotensin receptor blocker; ESRD: End-stage renal disease

Study (Author, Year)	Design / Setting	Population (N, Characteristics)	Intervention(s)	Comparator(s)	Follow-up Duration	Primary Cardiovascular Outcomes	Primary Renal Outcomes	Key Findings/Conclusions
Agarwal et al., 2022 [13]	Prespecified pooled analysis of 2 RCTs (FIDELIO-DKD + FIGARO-DKD), multicentre, double-blind	13,026 adults with T2D + CKD; broad spectrum of CKD (40% with eGFR >60 but albuminuria)	Finerenone + standard of care (RAAS inhibition)	Placebo + standard of care	Median 3.0 years (IQR 2.3–3.8)	Composite: CV death, non-fatal MI, non-fatal stroke, or HF hospitalization. HR 0.86 (95% CI 0.78–0.95); p=0.0018	Composite: kidney failure, sustained ≥57% decline in eGFR, or renal death. HR 0.77 (95% CI 0.67–0.88); p=0.0002	Finerenone reduced clinically important CV and renal outcomes across CKD spectrum in T2D. Safety similar, though hyperkalemia more frequent.
Sarafidis et al., 2023 [14]	Prespecified subgroup analysis of pooled phase III RCTs (FIDELIO-DKD + FIGARO-DKD), multicentre, double-blind	890 patients with stage 4 CKD (eGFR <30 ml/min/1.73 m²) out of 13,023 with T2D + CKD	Finerenone + standard of care (RAAS inhibition)	Placebo + standard of care	Median ~2.6 years (variable)	CV composite (CV death, nonfatal MI, nonfatal stroke, HF hospitalization): HR 0.78 (95% CI 0.57–1.07) → trend toward benefit	Kidney composite (kidney failure, sustained ≥57% eGFR decline, or renal death): effect protective up to 2 years, inconsistent afterward; finerenone reduced albuminuria and slowed eGFR decline	Finerenone provided a consistent CV benefit and slowed eGFR decline in stage 4 CKD. Kidney composite benefit less robust over time. Safety acceptable; hyperkalemia frequent but few discontinuations.
Bansal et al., 2024 [15]	Post hoc subgroup analysis of pooled phase III RCTs (FIDELIO-DKD + FIGARO-DKD), multicentre, double-blind	13,026 adults with T2D + CKD on optimized RAS inhibition; subgroups by age (<65, 65–74, ≥75 years) and sex (male, premenopausal female, postmenopausal female)	Finerenone + standard of care	Placebo + standard of care	Median 3 years	CV composite (CV death, nonfatal MI, nonfatal stroke, HF hospitalization): consistent benefit across age and sex; HRs: 0.94 (<65), 0.84 (65–74), 0.80 (≥75); males HR 0.86, females similar; more pronounced HF hospitalization reduction in males (Pinteraction=0.02)	Kidney composite (kidney failure, sustained ≥57% eGFR decline, renal death): benefit in <65 and 65–74, not ≥75; no significant heterogeneity by age or sex; consistent albuminuria reduction and slower eGFR decline	Finerenone improved CV and renal outcomes across age and sex subgroups. Greater HF hospitalization benefit in males. Safety consistent; hyperkalemia increased but discontinuation <3%.
Filippatos et al., 2025 [16]	Prespecified subgroup analysis of pooled phase III RCTs (FIDELIO-DKD + FIGARO-DKD), multicentre, double-blind	13,026 patients with T2D + CKD; 96.5% had hypertension; 9.6% had investigator-reported LVH on ECG at baseline	Finerenone + standard of care (RAAS inhibition)	Placebo + standard of care	Median ~2.5–3 years	CV composite (CV death, nonfatal MI, nonfatal stroke, HF hospitalization): overall benefit not modified by LVH (Pinteraction = 0.1075); greater benefit for HF hospitalization in the LVH subgroup (Pinteraction = 0.0024)	Kidney composite (kidney failure, sustained ≥57% eGFR decline, renal death): overall benefit not modified by LVH (Pinteraction = 0.1782)	Finerenone’s CV and kidney benefits were consistent regardless of baseline LVH. Patients with LVH gained additional protection against HF hospitalization. Safety similar; hyperkalemia higher with finerenone but discontinuations remained low.
Rossing et al., 2023 [17]	Prespecified subgroup analysis of pooled phase III RCTs (FIDELIO-DKD + FIGARO-DKD), multicentre, double-blind	13,026 patients with T2D + CKD; 944 (7.2%) on baseline GLP-1RA therapy	Finerenone + standard of care (RAS inhibition ± GLP-1RA)	Placebo + standard of care (RAS inhibition ± GLP-1RA)	Median ~2.5–3 years	CV composite (CV death, nonfatal MI, nonfatal stroke, HF hospitalization): benefit consistent with or without GLP-1RA; HR 0.76 (95% CI 0.52–1.11) with GLP-1RA vs HR 0.87 (95% CI 0.79–0.96) without; Pinteraction = 0.63	Kidney composite (kidney failure, sustained ≥57% eGFR decline, renal death): HR 0.82 (95% CI 0.45–1.48) with GLP-1RA vs HR 0.77 (95% CI 0.67–0.89) without; Pinteraction = 0.79	Finerenone reduced CV and kidney outcomes consistently regardless of GLP-1RA use. Greater UACR reduction with GLP-1RA (-38% vs -31%, Pinteraction = 0.03). Safety profile, including hyperkalemia risk, was consistent across subgroups.
Mentz et al., 2025 [18]	Post hoc subgroup analysis of pooled phase III RCTs (FIDELIO-DKD + FIGARO-DKD), multicentre, double-blind	12,990 patients with T2D + CKD; 51.6% on baseline diuretics (21.6% loop, 24.2% thiazide)	Finerenone + standard of care (RAAS inhibition ± diuretics)	Placebo + standard of care (RAAS inhibition ± diuretics)	Median ~2.5–3 years	CV composite (CV death, nonfatal MI, nonfatal stroke, HF hospitalization): finerenone reduced risk regardless of diuretic use; no effect modification (Pinteraction = 0.94)	Kidney composite (kidney failure, sustained ≥57% eGFR decline, renal death): finerenone benefit consistent regardless of diuretic use; no effect modification (Pinteraction = 0.55)	Finerenone reduced CV and kidney outcomes consistently, unaffected by baseline diuretic use. Hyperkalemia higher with finerenone vs placebo, but serious hyperkalemia leading to hospitalization or discontinuation was low across all subgroups.
van den Berg et al., 2022 [19]	RCT-based pharmacokinetic and time-to-event analysis (FIDELIO-DKD), double-blind, placebo-controlled	5734 patients with T2D + CKD; randomized 1:1 to finerenone 10 or 20 mg vs placebo	Finerenone 10–20 mg once daily, titrated + standard of care (RAS inhibition)	Placebo + standard of care	Median 2.6 years	CV outcomes were not the primary focus; exploratory assessment suggested benefit in reducing CV events consistent with overall trial findings	Kidney composite (kidney failure, sustained ≥57% eGFR decline, renal death): dose–exposure relationship showed efficacy plateau near 20 mg/day; maximal hazard reduction ~36%	Finerenone’s renal benefits were dose-dependent up to ~20 mg/day, with effects approaching saturation at this dose. No clinically relevant PK covariates altered outcomes. SGLT2 inhibitor co-use independently lowered CKD progression risk but did not modify finerenone’s effect.
Saglimbene et al., 2018 [20]	Multicenter RCT, open-label, blinded endpoint trial (LIRICO)	1243 adults with diabetes or CV risk factors + moderate/severe albuminuria	ACE inhibitor monotherapy, ARB monotherapy, or ACEi + ARB combination	Compared across the three treatment arms	Median 2.7 years	Primary CV composite (CV death, nonfatal MI, nonfatal stroke, CV hospitalization): no significant differences between groups	ESRD, doubling of serum creatinine, albuminuria, eGFR decline: no clear differences among groups	Trial underpowered (35% power, early stop). No significant differences in CV or renal outcomes between ACEi, ARB, or combination. ARB monotherapy had better tolerability (lower discontinuation) than ACEi or combination.

Quality Assessment

Table 2 presents the quality assessment of the included studies, showing that most RCTs and pooled analyses demonstrated a low overall risk of bias, particularly due to rigorous randomization, adherence to interventions, standardized outcome adjudication, and prespecified protocols. However, several subgroup analyses introduced concerns related to smaller sample sizes, reduced statistical power, and post hoc design, which limit the precision and generalizability of their findings. Exploratory pharmacokinetic modeling and early trial termination in one study [20] further contributed to some methodological weaknesses, with the latter being rated as high risk of bias. Overall, while the evidence base is strengthened by robust large-scale RCTs, caution is warranted when interpreting subgroup and secondary analyses due to inherent limitations.

**Table 2:** Quality assessment and risk of bias of included randomized controlled trials and subgroup analyses. RCT: Randomized controlled trial; CV: Cardiovascular; LVH: Left ventricular hypertrophy; GLP-1RA: Glucagon-like peptide-1 receptor agonist; CI: Confidence interval; N: Sample size (number of participants); PK: Pharmacokinetics

Study (Author, Year)	Study Type	Randomization Process	Deviations from Intended Interventions	Missing Outcome Data	Measurement of Outcomes	Selective Reporting	Overall Risk of Bias
Agarwal et al., 2022 [13]	Prespecified pooled analysis of 2 RCTs	Low risk – proper randomization, large sample	Low risk – double-blind maintained	Low risk – minimal missing data, balanced	Low risk – standardized CV/renal outcomes	Low risk – prespecified analysis, registry	Low
Sarafidis et al., 2023 [14]	Prespecified subgroup of pooled RCTs	Low risk – original RCTs well randomized	Low risk – intervention fidelity high	Some concern – smaller subgroup, reduced precision	Low risk – outcomes adjudicated	Low risk – prespecified subgroup	Some concerns
Bansal et al., 2024 [15]	Post hoc subgroup analysis of pooled RCTs	Low risk – based on RCT randomization	Low risk – intervention integrity preserved	Some concern – subgroup stratification may reduce power	Low risk – standardized outcome definitions	Some concern – post hoc nature	Some concerns
Filippatos et al., 2025 [16]	Prespecified subgroup analysis of pooled RCTs	Low risk – original randomization valid	Low risk – treatment adherence ensured	Some concern – only 9.6% with LVH → small subgroup	Low risk – blinded adjudication of CV/renal events	Low risk – prespecified subgroup	Some concerns
Rossing et al., 2023 [17]	Subgroup analysis of pooled RCTs	Low risk	Low risk	Some concern – only 7% using GLP-1RAs, wide CIs	Low risk – adjudicated outcomes	Low risk	Some concerns
Mentz et al., 2025 [18]	Subgroup analysis of pooled RCTs	Low risk	Low risk	Low risk – large N, balanced arms	Low risk – outcome assessment standardized	Low risk	Low
van den Berg et al., 2022 [19]	PK/time-to-event secondary analysis of RCT	Low risk – parent RCT randomized properly	Low risk	Some concern – modelling assumptions could bias	Low risk – outcomes derived from adjudicated trial data	Some concern – exploratory analysis	Some concerns
Saglimbene et al., 2018 [20]	Independent RCT, open-label, blinded endpoints	Some concern – open-label, potential performance bias	Some concern – adherence and discontinuations differed	Some concern – trial stopped early, underpowered	Low risk – blinded outcome adjudication	Low risk – prespecified outcomes	Some concerns

Discussion

Summary of Main Findings

Across eight high-quality randomized and post hoc analyses including more than 13,000 participants with T2D and CKD, finerenone consistently demonstrated significant cardiorenal benefits compared with placebo. In the prespecified pooled FIDELITY analysis, finerenone reduced the risk of the composite CV endpoint-CV death, nonfatal MI, nonfatal stroke, or hospitalization for heart failure-by 14% (HR 0.86, 95% CI 0.78-0.95; p=0.0018) over a median of three years. Parallel renal benefits were observed, with a 23% reduction in the risk of kidney failure, sustained ≥57% eGFR decline, or renal death (HR 0.77, 95% CI 0.67-0.88; p=0.0002). These effects were largely consistent across clinically relevant subgroups, including patients with stage 4 CKD, different age and sex categories, concomitant diuretic or GLP-1RA use, and those with LVH. Importantly, albuminuria reduction and slower eGFR decline were robust signals across studies. The main safety concern was hyperkalemia, which occurred in 10-26% of patients depending on subgroup, but discontinuation rates due to hyperkalemia remained below 3%, indicating acceptable tolerability.

Contextualization With Prior Literature

These findings reinforce and extend the current evidence base for MRAs in DKD [21]. Earlier MRAs such as spironolactone and eplerenone have shown renoprotective and cardioprotective effects, but their widespread use in CKD has been limited by high rates of hyperkalemia (up to 30-40% in stage 3-4 CKD) [22]. Finerenone’s non-steroidal structure and higher receptor selectivity confer distinct pharmacodynamic advantages; it binds the mineralocorticoid receptor with high affinity yet produces less conformational activation of pro-inflammatory and pro-fibrotic gene transcription in renal and cardiac tissue. This results in attenuated fibrosis, reduced oxidative stress, and improved endothelial function, mechanisms that likely underlie its superior safety and efficacy profile compared with steroidal MRAs. Current KDIGO 2022 guidelines and ADA/ESC recommendations [23] already endorse finerenone in patients with T2D, CKD, and albuminuria despite optimized renin-angiotensin system inhibition, a position strongly supported by these trial results. Furthermore, the consistent efficacy of finerenone regardless of concomitant SGLT2 inhibitor or GLP-1 receptor agonist use suggests complementary mechanisms of action. Notably, patients using both finerenone and GLP-1RA demonstrated a numerically greater reduction in albuminuria (-38% vs -31%; p-interaction=0.03), raising the prospect of additive benefit when combined with newer glucose-lowering agents.

Critical Appraisal of the Evidence

The evidence base supporting finerenone in DKD is strengthened by its foundation in large, well-conducted RCTs, notably the FIDELITY pooled dataset encompassing over 13,000 participants [13]. Outcomes were adjudicated and consistent across clinically relevant subgroups, reinforcing external validity. However, several limitations must temper interpretation. Many subgroup analyses, such as those stratified by age, sex, diuretic, or GLP-1RA use, were post hoc, increasing the risk of chance findings. Furthermore, patient populations were not fully representative: women comprised <30%, patients ≥75 years accounted for only ~15%, and those with stage 4 CKD formed a mere 7%. These gaps restrict generalizability, particularly in elderly and advanced CKD populations who are often at the highest risk. Additionally, median follow-up durations of ~2.5-3 years may be insufficient to fully capture long-term renal preservation, as kidney failure progression often spans decades. Finally, while hyperkalemia was manageable in trials, the safety profile requires confirmation in real-world practice where monitoring and adherence may be less rigorous.

Critical Insights

Several insights that extend the value of these findings emerge. From a real-world perspective, the highly selected trial populations with optimized RAAS inhibition may not mirror everyday practice, where polypharmacy, adherence barriers, and comorbidities may attenuate efficacy or amplify risks. Mechanistically, finerenone’s nonsteroidal structure and high receptor selectivity likely underpin its more favorable balance of efficacy and safety compared with older MRAs such as spironolactone, which exhibited hyperkalemia rates exceeding 30% in CKD cohorts [24]. Combination therapy represents another frontier: in subgroup analyses, concurrent GLP-1RA or SGLT2 inhibitor use did not diminish finerenone’s benefits, suggesting potential for additive or synergistic protection-though definitive trials powered for these interactions remain absent [25]. Notably, attenuated renal benefit in stage 4 CKD may reflect a ceiling effect of therapy once nephron loss is extensive, underscoring the need for adjunctive agents in late-stage disease. Finally, precision medicine signals, such as greater reductions in HF hospitalization among men and patients with LVH, highlight the possibility of tailoring therapy based on phenotypic markers, paving the way for more personalized cardiorenal protection strategies.

Research Gaps and Future Directions

Despite robust evidence from the FIDELITY program [13], several important gaps remain. First, there is a lack of head-to-head randomized trials directly comparing finerenone with older MRAs such as spironolactone or eplerenone in patients with T2D and CKD, a critical question, given differences in safety and cost. Second, most existing studies report median follow-up durations of 2-3 years, which are insufficient to determine whether finerenone meaningfully alters long-term renal trajectories beyond 5-10 years, especially with respect to dialysis initiation and kidney transplantation rates. Third, precision medicine approaches remain underexplored; biomarkers such as albuminuria reduction, LVH regression, and systemic inflammation markers may help identify subgroups with the greatest treatment benefit. Finally, implementation research in low-resource settings is urgently needed. Access to finerenone, affordability, and infrastructure for potassium monitoring are major barriers in many regions, and pragmatic trials in these contexts could ensure equitable translation of evidence into practice.

Practical and Policy Implications

Clinically, finerenone has the potential to be incorporated earlier in the treatment algorithm for patients with T2D and CKD, particularly in those with persistent albuminuria despite optimized RAAS inhibition. Early screening for albuminuria is therefore a crucial public health strategy, as emphasized by Agarwal et al. (2022) [13], yet remains underutilized in many healthcare systems. Broader adoption of finerenone will require careful planning for safety monitoring, particularly potassium surveillance, given the increased risk of hyperkalemia. This carries health system-level implications: laboratory infrastructure, digital monitoring tools, and workforce training must be strengthened to enable safe deployment at scale. Policymakers will also need to address cost-effectiveness considerations, as real-world uptake will be shaped by drug pricing, reimbursement, and comparative economic value against SGLT2 inhibitors and GLP-1RAs, which are also recommended in current international guidelines.

## Conclusions

Across eight high-quality studies including more than 13,000 patients, finerenone consistently demonstrated CV and renal protection in T2D with CKD, with benefits extending across diverse subgroups and clinical contexts. These findings reinforce its role as a cornerstone in the evolving landscape of cardiorenal therapeutics. This study uniquely integrates evidence from both large-scale RCTs and prespecified subgroup analyses, enabling a comprehensive appraisal of finerenone’s consistency across clinically relevant populations and concomitant therapies. However, uncertainties remain, particularly regarding its effectiveness in advanced CKD, underrepresented populations, and in head-to-head comparisons with older MRAs. By synthesizing subgroup analyses and highlighting gaps, this review provides clarity on where finerenone fits in current practice and outlines priorities for future research. Taken together, the evidence supports finerenone as a valuable addition to the standard of care, while reminding clinicians and researchers alike of the need for ongoing vigilance, precision, and innovation in the management of DKD.
